# Borderline personality disorder traits in adolescents with anorexia nervosa

**DOI:** 10.1002/brb3.2443

**Published:** 2021-11-22

**Authors:** Edna Lekgabe, Danielle Pogos, Susan M. Sawyer, Andrew Court, Elizabeth K. Hughes

**Affiliations:** ^1^ Department of Adolescent Medicine Royal Children's Hospital Melbourne Australia; ^2^ North Western Mental Health The Royal Melbourne Hospital Melbourne Australia; ^3^ Department of Paediatrics University of Melbourne Melbourne Australia; ^4^ Murdoch Children's Research Institute Melbourne Australia; ^5^ Centre of Adolescent Health Royal Children's Hospital Melbourne Australia

**Keywords:** adolescents, anorexia nervosa, borderline personality disorder, psychopathology

## Abstract

**Objective:**

To examine the correlation between eating disorder (ED) symptoms and borderline personality disorder (BPD) traits in a sample of adolescents with eating disorders.

**Method:**

There were 168 participants (*M*_age_ = 16.0 years; *SD *= 1.16) with a diagnosis of anorexia nervosa (AN) or Eating Disorder Not Otherwise Specified—AN type. Eating Disorder Examination (EDE) and the Borderline Personality Questionnaire (BPQ) were used to assess ED symptoms and BPD traits.

**Results:**

A total of 10 participants (6.6%) scored above the clinical cut‐off for a likely diagnosis of BPD. A positive correlation was observed between BPQ total score and EDE global (*r*_s _= 0.64, *p* < .001). There were also positive correlations between the BPQ self‐image and emptiness subscales and all EDE subscales. Similarly, the EDE eating concern subscale was correlated with all BPQ subscales.

**Discussion:**

Previous studies have demonstrated that some BPD traits (i.e., suicidality, impulsivity, anger) are co‐morbid with ED but the link with other BPD traits has been poorly studied in adolescents and those with AN. These findings indicate that while the prevalence of BPD in adolescents with AN may be relatively low, ED symptom severity is closely related to severity of BPD traits, particularly identity disturbance and feelings of emptiness.

## Abstract 2

Previous studies have demonstrated that some borderline personality disorder (BPD) traits (i.e., suicidality, impulsivity, anger) are co‐morbid with eating disorders (ED) but the link with other BPD traits have been poorly studied in adolescents and those with anorexia nervosa. This study aimed to examine the correlation between ED symptoms and all the BPD traits in a sample of adolescents.

## 1 INTRODUCTION

In the clinical setting, it is not uncommon to see borderline personality disorder (BPD) in patients with an eating disorder (ED). A review in the mid‐2000s demonstrated that the most prevalent co‐morbid personality disorder in patients with an ED was BPD; with a prevalence rate of 10% in restricting subtype of anorexia nervosa (AN), 25% in binge eating/purging subtype of AN, and 28% in bulimia nervosa (BN) (Sansone et al., 2005). The reverse was also true where the prevalence of an ED in patients with BPD was found to be high, varying from 10% to 54% (Chen et al., 2009; Zanarini et al., 2010). Research consistently shows that where an ED and BPD co‐exist, there is an increased level of distress, suicidal and non‐suicidal self‐injurious behaviors compared to having only one diagnosis (Ben‐Porath et al., 2009; Chen et al., 2009; Steiger & Stotland, 1996). While a co‐morbid personality disorder creates a more complex clinical picture, it also negatively impacts eating disorder treatment outcomes (Ben‐Porath et al., 2009; Wildes et al., 2011; Zeeck et al., 2007). As such, the underlying personality pathology needs to be considered so as to inform treatment options for both ED and BPD symptoms, especially for adolescents for whom the research in this area is limited.

BPD is typically described as a pervasive pattern of instability in interpersonal relationships, self‐image, and affect, along with marked impulsivity, beginning by early adulthood and leading to distress or impairment in social, occupational, or other areas of functioning (American Psychiatric Association, 2013). It is diagnosed in the presence of at least five of the nine symptoms: (i) avoidance of abandonment, (ii) unstable interpersonal relationships, (iii) identity disturbance, (iv) impulsivity, (v) recurrent suicidal behavior, (vi) affective instability, (vii) chronic feelings of emptiness, (viii) inappropriate and intense anger, and (ix) quasi‐psychotic ideation or severe dissociation (American Psychiatric Association, 2013). While the population prevalence of BPD is around 2%, it may be as high as 27% among patients seen in outpatient mental health clinics and about 15% among psychiatric inpatients (Sansone & Levitt, 2005). Notably, research on co‐morbid EDs and BPD has largely focused on adult populations. Despite BPD and EDs both known to emerge during adolescence, there is little research examining their relationship in adolescent samples.

The published data examining ED‐BPD co‐morbidity has also generally failed to explore the relationship between AN and BPD symptoms. It is notable that the majority of studies have focused on the examination of the relationship between BN and BPD; with the impulsive eating disorder behaviors encountered in BN appearing to echo the higher levels of anger, impulsivity, and non‐suicidal self‐injury symptoms typically seen in individuals with BPD diagnosis (American Psychiatric Association, 2013). While BPD symptoms have been identified as more strongly linked to BN than AN, the link with several other symptoms such as emptiness and fear of abandonment remain underexamined. Thus far there has only been one study which has examined the relationship between EDs in adolescents and each of the nine BPD symptoms (Miller et al., 2019). This study found correlations between BPD symptoms and both AN and BN symptoms with strong correlations for impulsivity with BN than AN symptoms.

Given the poor outcome at discharge and higher re‐admission rates seen in patients with co‐morbid BPD and EDs, a deeper understanding is needed of the relationship between BPD symptoms and EDs (Ben‐Porath et al., 2009; Wildes et al., 2011; Zeeck et al., 2007). This knowledge could potentially improve case formulation and help optimize treatment plans, especially within adolescents with AN for which there is limited existing data. We therefore aimed to examine the correlation between BPD traits and ED symptoms in a clinical sample of adolescents with AN.

## 2 METHOD

### 2.1 Setting

The study was conducted at a specialist eating disorder service at a tertiary pediatric hospital in Australia. All patients completed a standard set of measures as part of routine clinical care at the time of their initial assessment and diagnosis. The study was approved by the institutional ethics committee and consent was obtained to use this information for research. An eating disorder diagnosis was made by a psychiatrist following a multidisciplinary inpatient or outpatient assessment (Hughes et al., 2014).

### 2.2 Participants

Participants were 168 patients with a DSM‐IV diagnosis of AN restricting subtype (*n* = 90; 54%), AN binge/purge subtype (*n* = 3; 2%), or Eating Disorder Not Otherwise Specified—AN type (EDNOS) (*n* = 75; 45%). This latter group was included as they are now classified as Atypical AN in DSM‐5. Table 1 displays the participant characteristics.

**TABLE 1:** Mean and standard deviation of EDE and BPQ subscale scores (*N* = 168)

Measure	Mean (*SD*)	Range
PARTICIPANTS		
Age	16.0 (1.16)	14–18
BMI	17.35 (2.52)	11.38–26.18
Percent mBMI (%)	87.40 (11.81)	65–132
EDE		
Global EDE (4 subscales)	2.44 (1.74)	0–5.39
Restraint	2.70 (2.09)	0–6
Eating concern	1.91 (1.66)	0–5.8
Shape concern	2.69 (2.01)	0–6
Weight concern	2.43 (1.74)	0–5.39
BPQ^a^		
Total score	28.66 (16.70)	1–70
Impulsivity	1.17 (1.48)	0–7
Affect instability	5.76 (3.31)	0–10
Abandonment	2.39 (2.61)	0–9
Relationships	3.32 (2.10)	0–8
Self‐image	4.53 (3.06)	0–9
Suicide/self‐mutilation Self‐Mutilation	1.44 (1.91)	0–7
Emptiness	4.41 (3.36)	0–10
Intense anger	4.24 (3.22)	0–10
Quasi‐psychotic states	1.32 (1.52)	0–6

^a^ As participants were too young to drive, one item in the Impulsivity subscale regarding speeding was omitted and replaced with the participant's mean response to ensure scores were commensurate with other studies.

### 2.3 Measures

#### 2.3.1 The Borderline Personality Questionnaire

The Borderline Personality Questionnaire (BPQ) is a 80‐item (true/false) self‐report measure comprising nine subscales corresponding to the nine DSM‐IV BPD criteria (Poreh et al., 2006): impulsivity, affective instability, abandonment, relationships, self‐image, suicide/self‐mutilation, emptiness, intense anger, and quasi‐psychotic states. Scores range from 0 to 80, where a higher score indicates a higher level of pathology. A clinical cut‐off of 56 has been shown to predict BPD diagnosis with sensitivity of 0.68 and specificity of 0.90 amongst adolescents (Chanen et al., 2008). The BPQ has shown high test–retest reliability (Chanen et al., 2008). In this study, the Cronbach's alpha for the BPQ subscales ranged between 0.61 (impulsivity) and 0.88 (affective instability and intense anger). Given that patients in our sample were too young to drive, the item regarding speeding was omitted and replaced with the participant's mean response on the impulsivity subscale to ensure scores were commensurate with other studies. This has not been done in previous studies as samples have not included exclusively non‐driving participants.

#### 2.3.2 The Eating Disorder Examination

Eating disorder diagnosis was informed by use of the Eating Disorder Examination (EDE; version 16.0), a semi‐structured investigator‐based interview that measures ED psychopathology during the 4 weeks prior to assessment (Fairburn et al., 2008; Stice et al, 2000). The EDE yields four subscale scores: restraint, eating concern, weight concern, and shape concern. The subscale scores range from 0 to 6, with higher score reflecting greater symptom severity. Scores were averaged across subscales to form a global score measuring the overall severity of eating disorder psychopathology. Number of episodes of binge eating and purging in the previous 4 weeks (i.e., self‐induce vomiting, laxative use) were also recorded. The EDE has good reliability and validity and has been used in several studies, including in a population of adolescent girls (Berg et al., 2012; Mond et al., 2004; Wade et al., 2008). In this study, the Cronbach's alpha for the EDE subscales ranged between 0.76 (eating concern) and 0.92 (weight concern). The EDE was administered by trained research staff after supervised completion of at least 10 administrations by expert trainers.

### 2.4 Statistical analysis

The data were analyzed using Stata version 15.0. Missing values were replaced using expectation maximization for cases where there was less than 30% missing data for each subscale (Downey & King, 1998). Seventeen patients (10%) did not meet this criterion, and were excluded from the analysis. Means and standard deviations for each measure were calculated. Due to non‐normality, Bonferroni‐corrected Spearman's rho correlations were used to examine the relationship between ED symptoms and BPD traits. A Chi‐squared test was conducted to determine whether patients who scored above the clinical cut‐off on the BPQ (≥56) were more likely to binge eat or purge at least once a week compared with patients with low BPD traits.

## 3 RESULTS

Table 1 displays both the characteristics of the participants and scores on the EDE and BPQ. The participants ranged in age from 14 to 18 years (*M*_age _= 16.0 years; *SD* = 1.16) and were mostly female (90%). Body mass index (BMI) ranged from 11.38 to 26.18 (*M* = 17.35; *SD* = 2.52). The percent modified BMI (mBMI) ranged from 65 to 132% (*M* = 87.40; *SD* = 11.81). The EDE global score range 0 to 5.39 (*M *= 2.44; *SD* = 1.74) reflects the variation in self‐reported psychopathology usually seen in ED patients at presentation. Similarly, there was a large range seen in the BPQ scores, ranging from 1 to 70 (*M* = 28.66; *SD* = 16.70) with a total of 10 patients (6.6%) scoring above the clinical cut‐off.

Table 2 shows the correlations between the EDE and BPQ subscale scores. There was a large and significant positive correlation between the EDE global and BPQ total scores (*R*_s _= 0.64, *p* < .001). There were also several other significant positive correlations between various individual EDE and BPQ subscale scores, ranging from small to large. The strongest and most consistent were correlations between the BPQ self‐image and emptiness subscales and all EDE subscales. Likewise, the EDE eating concern subscale was correlated with all BPQ subscales.

**TABLE 2:** Spearman’s rho correlation matrix between BPQ and EDE subscales (*N* = 151)

BPQ subscales	Global EDE	Restraint	Eating concern	Shape concern	Weight concern
**Total score**	0.64***	0.49***	0.67***	0.60***	0.62***
**Impulsivity**	0.34**	0.27	0.31*	0.34**	0.32*
**Affective instability**	0.49***	0.40***	0.52***	0.44***	0.47***
**Abandonment**	0.52***	0.40***	0.56***	0.48***	0.51***
**Relationships**	0.45***	0.35**	0.53***	0.37***	0.41***
**Self‐image**	0.69***	0.55***	0.69***	0.66***	0.69***
**Suicide and self‐harm**	0.44***	0.38***	0.42***	0.43***	0.41***
**Emptiness**	0.60***	0.45***	0.61***	0.60***	0.56***
**Anger**	0.34**	0.28	0.34**	0.32*	0.35**
**Quasi‐psychotic states**	0.45***	0.37***	0.46***	0.41***	0.43***

* Significant at *p* < .05.

** Significant at *p* < .01.

*** Significant at *p* < .001.

In order to facilitate clinical interpretation of the presence of regular binge/purge episodes in young people with likely BPD, a Chi–squared test was used to determine whether patients who scored above the clinical cut‐off on the BPQ were significantly more likely to binge/purge at least once a week for the previous 4 weeks compared to patients who scored below the BPQ clinical cut‐off. Patients scoring about the clinical cut‐off on the BPQ were more likely to engage in both regular binge episodes, *χ* ^2^ (2, *N* = 151) = 8.97, *p *= .011, *φ *= 0.24, and regular purging, *χ* ^2^ (2, *N *= 151) = 28.18 *p *< .001, *φ *= 0.43.

## 4 DISCUSSION

This study indicated that while the prevalence of BPD in adolescents with AN may be relatively low (around 7%), ED symptom severity is closely related to severity of BPD traits, particularly disturbance in self‐image and feelings of emptiness. While previous studies have demonstrated that some BPD traits (i.e., suicidal behaviors, impulsivity, anger) are co‐morbid with BN and AN‐binge/purge type, the link with other BPD traits have been poorly studied, especially in adolescents. Given the complexity that co‐morbid BPD lends to clinical interventions and outcome, more research into the interplay between symptom types is needed (Wildes et al., 2011; Zeeck et al., 2007). In this study, we set out to investigate the correlation between ED symptoms and all nine individual BPD traits in an adolescent population. To our knowledge, this is only the second study to investigate this relationship.

We found that there was a strong and significant relationship between ED symptoms and BPD symptomatology overall. In particular, adolescents who reported self‐image disturbance and feelings of emptiness were most likely to have restrictive eating, eating concern, and dissatisfaction with shape and weight. Of importance, the BPQ self‐image subscale items measure unstable self‐image or sense of self rather than body image. This data supports the accepted psychopathology underpinning AN, which describes a perfectionism and an overvalued idea that can become central to their sense of identity, leaving the individual with virtually no sense of themselves outside of the ED (Higbed & Fox, 2010). It is perhaps not surprising then that unstable self‐image was associated with more efforts to restrict, greater eating concerns, and poorer body image. Although this study could not examine a causal relationship, we could speculate that unstable self‐image is a predisposing factor for the development of restrictive eating. This is in line with Fairburn's transdiagnostic model which places low self‐esteem as central to the development of EDs (Fairburn et al., 2003).

From a personality profile perspective, adolescents scoring above the clinical cut‐off for a BPD diagnosis, were more likely to report binge eating and/or purging at least once a week for the previous 4 weeks. This was not unexpected given that these behaviors are more typical of those with BN which has previously shown to be frequently co‐morbid with BPD (Claes et al., 2015; Sansone et al., 2005). It has been suggested that the commonality may be amongst individuals with high impulsivity and poor emotion regulation (Racine & Horvath, 2018). If so, this may have important implications for treatment planning with individuals with this presentation profile (Thompson‐Brenner et al., 2008; Westen & Harnden‐Fischer, 2001).

There are several emerging options for patients presenting with co‐occurring ED and BPD symptoms; however, the most well established is dialectical behavior therapy (DBT). Although not first‐line treatment for EDs, the emotional regulation skills taught in DBT can be used to target the individual BPD symptoms most relevant to maintaining the ED psychopathology. Recent studies have shown encouraging results with the use of DBT in treating co‐morbid EDs and BPD (Bankoff et al., 2012; Navarro‐Harro et al., 2018).

One of the limitations of this study is that the group under study is young and personality traits may still be developing. It is therefore possible that BPD symptoms are confounded by transient developmental states. Other limitations include, the self‐report nature of the BPQ and EDE, the small sample size and correlational nature of the study. Per se, we are unable to examine a causal relationship between BPD and ED psychopathology. However, this study does highlight relevant associations between the presence of various BPD traits (i.e., poor self‐image) with ED symptoms in adolescents, which may be a useful for conceptualizing the illness and tailoring treatment of the eating disorders in order to improve outcomes and reduce relapse.

### PEER REVIEW

The peer review history for this article is available at https://publons.com/publon/10.1002/brb3.2443

## FINANCIAL SUPPORT

The Murdoch Children's Research Institute is supported by the Victorian Government's Operational Infrastructure Program. This work was supported by a grant from the Baker Foundation.

## CONFLICT OF INTEREST

The authors have no conflict of interest to declare.

## Data Availability

The data that support the findings of this study are available from the corresponding author upon reasonable request.
